# Potential of Entomopathogenic Nematode HbSD as a Candidate Biocontrol Agent against *Spodoptera frugiperda*

**DOI:** 10.3390/insects14010002

**Published:** 2022-12-21

**Authors:** Yuan Chen, Haibo Long, Tao Jin, Zhengqiang Peng, Yanfang Sun, Tuizi Feng

**Affiliations:** 1 Key Laboratory of Integrated Pest Management on Tropical Crops, Ministry of Agriculture and Rural Affairs, Environment and Plant Protection Institute, Chinese Academy of Tropical Agricultural Sciences, Haikou 571101, China; 2 Hainan Key Laboratory for Monitoring and Control of Tropical Agricultural Pests, Haikou 571101, China

**Keywords:** entomopathogenic nematode, *Spodoptera frugiperda*, Heterorhabditidae, biological control

## Abstract

**Simple Summary:**

*Spodoptera frugiperda* is a highly destructive and polyphagous pest that causes severe damage to various crops. Great efforts have been devoted to developing more effective and friendly strategies to minimize losses caused by *S. frugiperda*. In the current study, the infective capabilities of entomopathogenic nematode species HbSD, belonging to *Hetrerorhabditis bacteriophora*, were evaluated under laboratory, greenhouse and field conditions. The results showed that HbSD was highly lethal to *S. frugiperda* under laboratory/greenhouse conditions; however, the efficacy of HbSD decreased in the field trials. To better exploit HbSD as a biological agent, more efforts need to be carried out in future field work, including the optimization of production, formulation and application technology and other diverse factors.

**Abstract:**

*Spodoptera frugiperda* is a highly destructive and polyphagous pest that causes severe damage to various crops, especially maize. The wide use of chemical insecticides to control *S. frugiperda* results in resistance against commonly used chemicals and resistant mutations will expand in populations accompanied by a spread to vulnerable areas. Consequently, more effective and friendly strategies must be explored to minimize losses caused by *S. frugiperda*. Entomopathogenic nematodes (EPN) are good candidates for the biological control of different species of insect pests, including *S. frugiperda*. In the current study, the infective capabilities of the EPN species HbSD, belonging to *Hetrerorhabditis bacteriophora*, were evaluated against *S. frugiperda* under laboratory, greenhouse and field conditions. In laboratory assays, HbSD was highly virulent against 3rd/5th instar larvae, which was related to HbSD concentration and exposure durations. In greenhouse assays, spraying aqueous HbSD also showed good performance in killing larvae on maize leaves. However, the virulence of HbSD decreased in field trials where many adverse factors affecting survival and efficacy were encountered by HbSD. Overall, our study provides an alternative EPN for the biological control of *S. frugiperda* with the potential to be developed as a sustainable option for efficient pest management.

## 1. Introduction

The fall armyworm (FAW), *Spodoptera frugiperda* (J.E. Smith) (Lepidoptera: Noctuidae) is native to tropical and subtropical regions of America and has rapidly spread to over 70 countries in Africa, Asia, Australia and Europe since 2016 [1]. FAW causes severe damage to the leaves and stems of at least 353 species in 76 plant families [2]. According to the differences in host preference, FAW has been categorized into two sub-populations, the “rice-strain” (R-strain) and the “corn-strain” (C-strain) [3]. The corn-strain haplotype mainly feeds on corn, cotton and sorghum, while the rice-strain haplotype invades rice and pastures [4]. Due to its high capacity of long-distance migration and adaptation, FAW continues spreading into new territory and is widely regarded as the most damaging pest of global economic importance.

In China, FAW was first reported in Yunnan Province in December 2018 and spread rapidly to 16 provincial regions in Southern China within 4 months [5]. By 2020, it has invaded into 27 provinces, covering tropical, subtropical and temperate climate zones in China. A wide range of potential distribution by FAW has been forecast in China, and the centers of the potential distribution areas have mainly moved northward [6]. Previous genetic identification confirmed that the FAW population invading the Yunnan province belonged to “corn-strain” type [7]. China is the second largest corn producer in world, and the main corn-growing areas are also within the suitable habitat range for FAW. According to field inspection data, more than 1.125 million hectares of cropland were infected by FAW in 2019 and 1.278 million hectares in 2020 [5]. To date, FAW has been reported in all but five provinces in the northwest and northeast regions, covering about 13 million hectares of corn-growing areas in China [8]. Consequently, the Chinese central government has remained vigilant since it was first detected and are paying great attention to preventing the further invasion and spread of FAW.

FAW infections cause high yield losses that result in significant economic impacts. In the USA, around USD 300 million yield lost was caused by FAW, rising to USD 500 million or more in outbreak years [9]. In Africa, it was estimated that up to 17.7 million tons of maize was infected by FAW annually and resulted in losses valued from USD 1.1 to 4.7 billion [10]. Due to the rapid global invasion capacity and devastating damages to agricultural crops, there is a pressing need for the effective management of FAW. Chemical insecticides are widely used and function as the most effective weapon for FAW management. However, the misuse or overuse of chemical insecticides have resulted in evolved resistance in the FAW population. It was reported that FAW showed resistance to 45 active ingredients (https://www.pesticideresistance.org/display.php?page=species&arId=200, accessed on 12 November 2022). The global invasion of FAW was accompanied by the spread of resistance mutations against synthetic insecticides in invaded areas [1]. In China, the invading FAW populations carried resistance to organophosphate and pyrethroid pesticides [11]. As resistance to insecticides is the main challenge in pest control, cultural and biological strategies must also be adopted to fight FAW. For FAW management, the applications of extracts and metabolites from plants, such as limonoids and azadirachtin, is considered to be effective, cost-efficient, environmentally friendly and without negative effect to consumers [12]. Biological controls using microbials, including entomopathogenic fungi, viruses, nematodes and bacterium, are now important components in integrative FAW management, all of which represent sustainable and promising strategies [13].

Nematodes that parasitize insects, known as entomopathogenic nematodes (EPN), have been considered as one of the most effective biological agents against pests. EPNs are lethal parasites of insect hosts which release symbiont bacteria, such as *Xenorhabdus* and *Photorhabdus*, after invading the body cavity of host insects [14,15]. Of all the EPNs, Steinernematidae and Heterorhabditidae families draw the most attention, and about 100 species of *Steinernema* and 21 species of *Heterorhabditis* had been identified on different continents of the world prior to 2020 [14,16]. Due to their attributes of having a broad host range, a rapid speed of kill, compatibility with chemical pesticides and being ecologically friendly, EPNs are employed as an alternative strategy for insect pest control [17,18]. The virulence of various EPNs were evaluated in a laboratory or field against species of *Ceratitis capitata*, *Popillia japonica*, *Leucopholis lepidophora* and so on [19,20,21]. In general, most of the applications of EPNs were aimed at soil containing pest insects. For foliar insects, promising results were found in laboratory and glasshouse trials, including against *Coleoptera*, *Diptera*, *Hemiptera*, *Hymenopera*, *Lepidoptera* and *Thysanoptera* [22]. Considering the high application potential to suppress larval populations, at least eight *Steinernema* species and five *Heterorhabditis* species have been commercialized [23].

There have also been a series of reports on EPN screening FAW as a target, which indicates the potential of their practical application to control FAW [24,25,26].

In a previous study, we identified one EPN belonging to *H. bacteriophora* strain SD, named as HbSD [27]. To further explore the applied potential of HbSD, we evaluated its virulence on FAW under different growing conditions, including laboratory, greenhouse and field. The obtained results will further illuminate the efficacy of HbSD and help to provide an alternative biological agent to control FAW.

## 2. Materials and Methods

### 2.1. S. frugiperda and EPN Collection and Culturing

*S. frugiperda* larvae were collected from a cornfield in Wenchang, Hainan province, China. The caterpillars were maintained at feeding conditions: temperature 26 ± 0.5 °C, relative humidity 65 ± 5% and a photoperiod of 16:8 h light/dark cycle. The larvae were fed with an artificial diet, as described previously [28]. The adult moths were fed with a 10% honey water solution.

The EPN strain HbSD was first collected from field near Danzhou City, Hainan Province, China [27]. It was kept in our laboratory and were reared in vivo on *Tenebrio molitor* larvae, as previously described [29]. The infective juveniles (IJs) of EPNs were collected via White traps in Petri plates (120 mm) [30]. The harvested IJs were stored in distilled water in 225 mL Corning cell culture flasks at 10 °C in the dark before use within 2 weeks. Before each experiment, the IJs were acclimatized to room temperature for an hour and the movement of IJs was observed under a stereomicroscope. Only active IJs were used to prepare the IJ stock suspensions for all trials.

### 2.2. Virulence of HbSD against S. frugiperda Larvae in Petri Plate Bioassay

The bioassay arena was set up in a glass Petri plate (90 mm diameter) that was lined with two sterile filter papers. Then 1 mL of HbSD suspension containing approximately 60, 120, 250, 500, 1000, 2000 and 5000 IJs was added to each dish, while the control plates were added 1 mL of distilled water. A total of 20 caterpillars at the 3rd or 5th developmental stages were placed in each arena with the artificial diet. The concentration of IJs in each plate were 3, 6, 12, 25, 50, 100 and 250 IJs per larva, respectively. Each treatment contained 5 replications. Finally, each Petri dish was monitored for 24, 36 and 48 h post-application treatment and larval mortality was calculated according to the number of dead larvae. The insect cadavers were dissected under stereomicroscope. The mortality data were corrected using Abbott’s formula [31], as seen in Equation (1). The median lethal concentration (LC50) value was determined for the 3rd larvae when treated for 36 h with HbSD.

(1) $$\mathrm{Control}\;\mathrm{mortality}\;\left(\%\right)=\frac{\mathrm{survival}\;\mathrm{in}\;\mathrm{the}\;\mathrm{control}-\mathrm{survival}\;\mathrm{in}\;\mathrm{the}\;\mathrm{treatment}}{\mathrm{survival}\;\mathrm{in}\;\mathrm{the}\;\mathrm{control}}\;\times 100$$

### 2.3. Efficacy of HbSD against S. frugiperda Larvae in Potted Soil Bioassay

The plastic pots (8.5 cm diameter) were filled with 200 g of sandy loam soil. Each pot was planted 3 maize seedlings for 20 days before used. Suspensions of HbSD containing 5000, 10,000 and 20,000 IJs were added into a watering can with a misting spray nozzle. A total of 500 mL EPN spray liquid was first applied for each pot. Then 10 larvae at the 3rd instar were released onto the maize seedlings in each pot. For the control, the equal volume of water without IJs was sprayed onto the maize seedlings. The number of dead larvae was determined after treatment for 48 h and 72 h. Each treatment consisted of 5 replicates as each pot represents a replicate. The control mortality was calculated by Abbott’s formula as described above.

### 2.4. Field Trial

At the beginning of 2022, the field experiments were conducted at a corn growing area in Wenchang, Hainan. All the experiments were carried out with commercial corn seeds “Bai Ru Yu” (Hainan Lv Chuan Seed Co., Ltd., Haikou, Hainan, China) in the experimental plots. The seeds were sown in Dec 2021 and used for field experiments after one month. 

In this experiment, three treatments for HbSD were set, as the final concentrations were about 10,000/25,000/50,000 IJs per plant. Negative control plants were sprayed with the same volume of water. Plants sprayed with indoxacarb SC (FMC corporation, Philadelphia, PA, USA), which is known to be a promising foliar insecticide with strong field activity against Lepidoptera, were set as the positive control [32]. The final concentration of indoxacarb SC applied as the insecticide was 15 mL/mu. For each treatment, 50 corn seedlings were included and 4 replicates were randomly distributed in test plots. HbSD nematodes were harvested within 2 weeks prior to field application and the HbSD suspensions were prepared in sterile water. The density of IJs were counted and adjusted as required for field concentration per treatment. When applied EPN or insecticide suspensions, a knapsack electric sprayer was used with a spraying pressure of ~0.3 Mpa. A total of 16 L suspensions were sprayed for each treatment, and the equipment was shaken thoroughly during application.

To assess the severity level caused by *S. frugiperda*, we referred to the grading criteria of corn leaves, modified as previously described [33,34]. The disease progression of each plant was determined by the percentage of infected leaves. As shown in Table 1, the damage severity of corn plants was categorized from Grade 0 (healthy) to Grade 5 (severe damage). We investigated all the corn plants in each treatment and categorized them into one of the six levels of severity after spraying for 5 days and 20 days. Then the damage index (DI) of corn leaves was calculated using Equation (2). The control efficacy for each treatment was calculated according to Equation (3).

(2) $$\mathrm{DI}=\frac{\sum \left(\mathrm{Number}\;\mathrm{of}\;\mathrm{leaves}\;\mathrm{of}\;\mathrm{each}\;\mathrm{grade}\;\times \mathrm{Disease}\;\mathrm{grade}\right)}{\mathrm{Maximum}\;\mathrm{disease}\;\mathrm{grade}\;\times \;\mathrm{The}\;\mathrm{number}\;\mathrm{of}\;\mathrm{total}\;\mathrm{plants}}\times 100$$

(3) $$\mathrm{Control}\;\mathrm{efficacy}\;\left(\%\right)=\frac{\mathrm{DI}\;\mathrm{of}\;\mathrm{control}-\mathrm{DI}\;\mathrm{of}\;\mathrm{treatment}}{\mathrm{DI}\;\mathrm{of}\;\mathrm{control}}\times 100$$

### 2.5. Statistical Analysis

In all experiments, statistical analyses and graphs were finished with OriginPro 2021 software version 9.8 (OriginLab, Northampton, MA, USA). All data obtained in this study were imported into the OriginLab workbook and statistical analyses were conducted, including the mean and standard errors calculation of biological replicates. One-way ANOVA was adopted to compare difference of means between treatments using Tukey’s HSD test, with the P-value set as 0.05. The LC50 value was calculated by a nonlinear curve fit to the Levenberg–Marquardt equation using OriginPro 2021 software.

## 3. Results

### 3.1. Pathogenicity of HbSD to S. frugiperda Larvae under Laboratory Conditions

**Figure 1:**
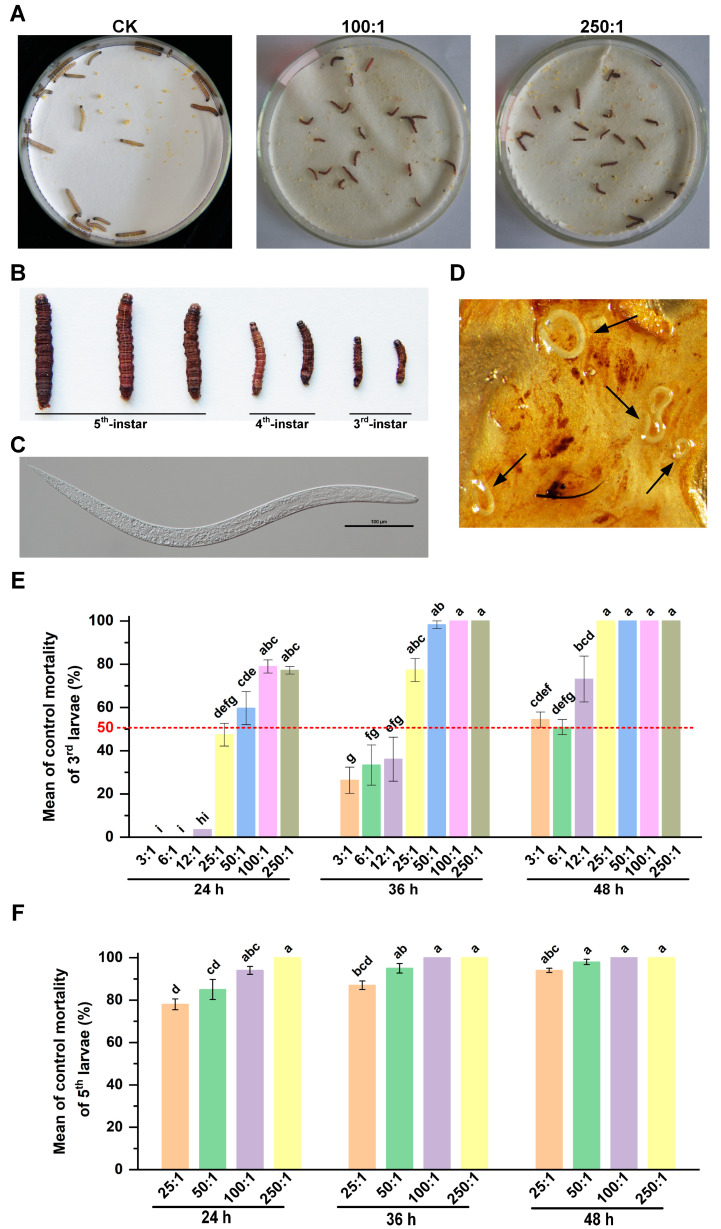
HbSD efficacy against *S. frugiperda* in Petri plate bioassays. (**A**) Representative plates containing 3rd instar larvae of *S. frugiperda* treated with water and HbSD suspensions at 48 h post-treatment. (**B**) The cadavers of *S. frugiperda* caused by HbSD infection for 48 h. (**C**) Observation of infective juvenile of HbSD under microscope. (**D**) The body contents inside the cadaver of *S. frugiperda* under stereomicroscope. The arrows indicate HbSD nematodes entered the insect body. (**E**,**F**) The mean control mortality (% ± SE) of 3rd (**E**) and 5th (**F**) larval stages of *S. frugiperda* treated with HbSD in Petri plates. The significant differences are exhibited by lowercase letters (*p* < 0.05) according to Tukey’s HSD tests.

In this assay, the larval mortality of *S. frugiperda* was assessed at 3rd and 5th development stages, as EPNs typically infect late-stage larvae. As shown in Figure 1, the EPN species of HbSD exhibited lethal capabilities towards *S. frugiperda*. After being infected with HbSD, the entire bodies of the killed insects turned brick-red (Figure 1A,B). We first observed the morphology of HbSD under microscope before infection (Figure 1C). When we dissected the insect cadavers under a stereomicroscope, the body contents were viscous and HbSD nematodes had entered the insect body (Figure 1D). HbSD was confirmed to be highly virulent to *S. frugiperda*, as only 3 IJs per 3rd stage larvae were enough to cause 54.39% mortality after being treated for 48 h. After we calculated the mortality, we found that along with the increase in EPN concentration and exposure time, the corrected mortality caused by HbSD was also increased (Figure 1E,F). For 3rd instar larvae, a series dosage of IJs of HbSD was applied in order to evaluate the virulence. In the first 24 h, at least 12 IJs were needed to cause the caterpillars to die. The control mortalities ranged from 26.32% to 100% when treated with 3 to 250 IJs per larvae for 36 h. This indicates that EPN densities are essential, as lower numbers of IJs needed more time to infect and reproduced inside the insect’s body (Figure 1E). In addition, the LC50 value was 17.23 IJs per larvae for 3rd stage larvae when treated by HbSD for 36 h. Notably, our results showed that only 3 IJs per larvae was able to cause a more than 50% mortality rate after exposure for 48 h, which confirmed the highly virulent nature of HbSD for *S. frugiperda*. For 5th instar larvae treated for 24 h by HbSD, the mortality was 78.33% to 100%. This indicated that the larvae at the 5th stage were more susceptible to HbSD in the first 24 h as compared to 3rd stage larvae. When the concentration of HbSD reached more than 100:1 per larva, 100% mortality was caused at 36 h and 48 h post-treatment. In addition, the pathogenicity of HbSD was related to larval stages, as only 25 IJs per larva at 3rd stage were enough to cause 100% mortality at 48 h post-treatment, while at least 100 IJs per larva were needed for 5th stage larvae. In conclusion, HbSD exhibited a good ability to kill *S. frugiperda* larvae under laboratory conditions.

### 3.2. Effect of HbSD against S. frugiperda Larvae in Potted Soil Bioassays

**Figure 2:**
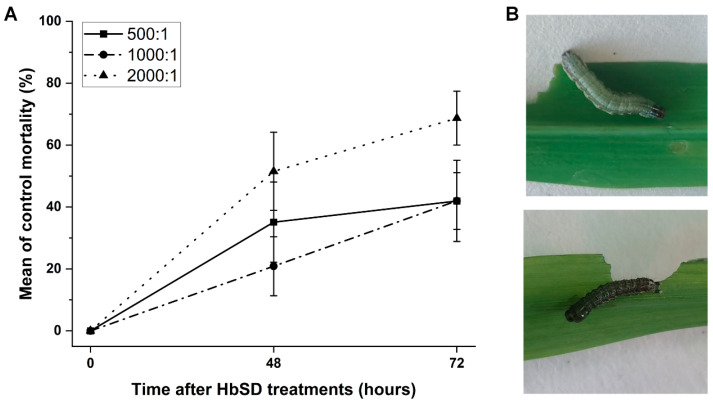
HbSD efficacy against *S. frugiperda* in potted soil bioassays. (**A**) The control mortality caused by HbSD infection at concentrations of 500:1, 1000:1 and 2000:1 per larva when treated for 48 h and 72 h. (**B**) The alive (above) and dead (below) larvae picked from corn leaves after treating with water or HbSD for 72 h.

To further assess the lethal effects of HbSD against *S. frugiperda*, the mortality rates of 3rd instar larvae were determined in potted soil bioassays. On the whole, a higher concentration of HbSD and longer exposure time resulted in higher mortality rates. The highest mortality was caused by HbSD at the concentration of 2000:1 per larva, with a mortality of 51.56% at 48 h and 68.72% at 72 h (Figure 2A). At lower concentration of HbSD, such as 500:1 and 1000:1, the mortality rates at 72 h were nearly the same (~40%). This indicated that the opportunities for *S. frugiperda* to encounter EPNs were limited by the concentration. The dead larvae of *S. frugiperda* picked from corn leaves were a different color compared to living larvae (Figure 2B), indicating that the death of the insects resulted from HbSD infection.

### 3.3. Efficacy of HbSD against S. frugiperda Applied in Field

**Figure 3:**
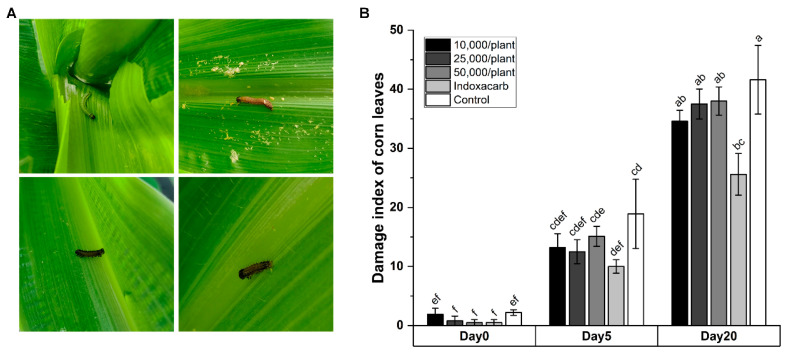
HbSD efficacy against *S. frugiperda* in field applications. (**A**) The alive (upper left corner) and dead larvae observed on corn leaves in field plots. (**B**) The damage index of corn leaves observed in field plots treated with HbSD suspensions, insecticide (specifically indoxacarb) and water. The significant differences are exhibited by lowercase letters (*p* < 0.05) according to Tukey’s HSD tests.

To evaluate the applied potential of HbSD to control *S. frugiperda* in the field, we conducted a field trial in a maize growing plot at the beginning of 2022. After spaying HbSD solutions for 20 days, brick-red cadavers were found on inner corn leaves grown in treatment plot (Figure 3A). As most of the dead larvae were 2nd or 3rd instar, these larvae had perhaps just hatched from pupae and climbed out from the inner leaves to seek food, where they encountered and were infected by the IJs of HbSD. Unfortunately, no statistically significant difference of the damage index of corn leaves among treatments was observed compared with the control plots, while the plots treated with indoxacarb insecticide exhibited a significant difference (*p* < 0.05; Figure 3B). For each treatment, the control efficacy was 43.18% (10,000/plant), 51.20% (25,000/plant), 25.17% (50,000/plant) and 89.00% (indoxacarb) after 5 days. After sprayed for 20 days, the control efficacy decreased to 20.23% (10,000/plant), 10.93% (25,000/plant), 9.47% (50,000/plant) and 62.5% (indoxacarb). The low efficacy and sustainability of HbSD in field application could possibly be attributed to EPN survivability, temperature and humidity conditions and spraying efficiency.

## 4. Discussion

FAW is notorious for its wide host range, high reproductive and dispersal capacity and ability to cause severe damage to crop worldwide. Among the host plants, maize is the most favored by FAW larvae and can be struck during the vegetative and reproductive or flowering phase. FAW larvae are also able bore into maize ears, stems and cobs, which make them more concealed and harder to spot in time [35]. To date, the management of FAW by chemical control is the most effective and prevalent strategy. To minimize the adverse effects of the misuse or overuse of chemical insecticides, biological alternatives against FAW are constantly being explored. Thus, EPN species have garnered significant attention as an environmentally sustainable biocontrol agent in pest control. As EPNs are predominantly isolated from soil habitats, they have been extensively exploited to suppress soil-dwelling insect pests in agricultural fields. Recently, the foliar applications of EPN biocontrol have been reported for the targeting of several lepidopteran pests, such as Tortricidae, Plutelidae, Gelechiidae and Erebidae [36,37,38,39,40]. To widen the utilization of EPN for biocontrol, isolating and screening more EPN species/strains that meet the efficacy requirements is the first pivotal step. For this reason, we evaluated the efficacy of HbSD, a novel EPN strain isolated by our laboratory previously, against FAW under different conditions.

In the current study, we confirmed that HbSD had good performance in controlling *S. frugiperda* in both laboratory and greenhouse conditions, which was related to concentration of HbSD solutions, exposure time and FAW developmental stage. In laboratory bioassays, it only took 36 h to attain 100% mortality when infected with HbSD at the concentration of 100 or 250 IJs per 3rd stage larva of *S. frugiperda*. Notably, infection for 24 h was enough to cause the 100% mortality of *S. frugiperda* at a concentration of 250 IJs per 5th stage larva. Although it has been reported that *H. bacteriophora* was virulent in many insect species, including *S. frugiperda*, its virulence and killing speed varied among strains within EPN species [41]. For instance, *H. bacteriophora* collected from Daedong Tech (Daegu, Korea) caused ~60% mortality in 3rd instar larvae and ~50% mortality in 5th instar larvae at a concentration of 50 IJs per larva for 72 h post-treatment [26]. *H. bacteriophora* HP88 caused 65%, 95% and 85% mortality at concentrations of 100, 250 and 500 IJs per 5th instar larvae after inoculation for 4 days [24]. As shown in previous studies, two strains of *H. indica* showed differential mortality against 3rd stage larvae, with LC50 values of 21.65 and 48.91 IJs/larva, respectively [42]. Lalramnghaki et al. studied four EPN species on *S. frugiperda*, with the LC50 value ranging from 20.26 to 35.08 IJs/larva at 72 h post-incubation for 3rd larval instars [43]. In our study, the LC50 value of HbSD was 17.23 IJs per 3rd larvae at 36 h post-incubation, which indicated the high rate of larvicidal activities against *S. frugiperda*. In pot assays, spraying HbSD suspension on maize leaves also exhibited high lethal effects on FAW larvae at concentrations of 2000 IJs per 3rd stage larva, which encouraged its use on plant foliage.

EPNs are considered to be one of the most promising biocontrol agents in insect pest control, while its application for broader utilization in the field still faces great challenges. Indeed, there are many obstacles in the translation of the good efficacy in laboratory experiments into success in field. As shown in previous studies, the larval and adult *Heteronychus arator* were susceptible to *S. carpocapsae* in laboratory assays, while field tests using *S. carpocapsae* against adults were unsuccessful [44]. Similar results were obtained in tests of the *Steinernema* species against the carob moth *Ectomyelois ceratoniae* [45]. In our study, HbSD faced the same issues in field trial when attempting to control FAW, as the control efficacy was low and was unqualified to be an effective biocontrol inoculant. After 5 days post-treatment, the control efficacy of each treatment ranged from 25–51%, while it decreased to 9% to 20% after 20 days post-treatment. This indicates that the efficacy of HbSD was not stable over time. The difference between applications in laboratory/greenhouse and field trials was first caused by factors affecting EPN survival and infection. It can be concluded that the success of EPN application for foliar pests depends on abiotic conditions, such as moisture and temperature and biotic conditions, for instance the pathogenicity and foraging strategy of nematode species [46]. Compared with treating soil-dwelling pests, EPN application on foliar environments is more vulnerable to unfavorable conditions and less successful than soil-based applications [47]. After being sprayed on maize leaves, IJs of HbSD were exposed to adverse environmental conditions, including temperature, ultraviolet radiation and the risk of desiccation [48]. Therefore, EPNs are usually applied in high numbers, up to billions of nematodes, to increase the chances of finding host insects. Other efforts to increase the survival time of EPNs after aboveground application, such as mixing with a surfactant and polymer, successfully improved control efficacy via increased leaf coverage [49]. Secondly, the time of EPN application is also crucial for the effective control of foliar pests. The results collected from Petri plate bioassays indicated that the efficiency of HbSD was dependent on host larval stage, as different mortalities were observed between the 3rd and 5th instar under the same EPN concentration and exposure time. It is likely that if the application time coincides with the susceptible pest life stages, the control effects will increase. In addition, application technology is another key point to improve HbSD efficacy in field. The efficiency of equipment used for spray EPN formulations is determined by nozzle/dripper type, volume, agitation, pressure and recycling time [50]. Overall, identifying highly infectious EPN strains, such as HbSD in this study, cannot fully meet the requirements of FAW field control, and thus, efforts to improve the tolerance of aboveground abiotic factors are also required. For this reason, the formulation and application technology of HbSD needs to be improved in future work to enhance its efficacy in controlling FAW in field trials.

## 5. Conclusions

In the present study, we carried out a comprehensive assessment of control efficacy of HbSD against *S. frugiperda*. These results confirmed that HbSD was highly lethal to S. *frugiperda* under laboratory/greenhouse conditions, which examined the feasibility of using HbSD to control *S. frugiperda*. Unfortunately, when we sprayed aqueous HbSD on maize leaves in field trials, the damage index of maize leaves showed no significant difference compared with negative controls. Environmental conditions affecting EPN survival and efficacy hinder their broader application against foliage pests, such as *S. frugiperda*. To better exploit HbSD as a biological agent, more efforts are needed to improve future field work, including the optimization of production, the formulation and application of technology and other diverse factors.

## Figures and Tables

**Table 1:** The grading criteria of corn for *S. frugiperda* infection.

**Grade**	**Grading Standard**
0	Healthy
1	Leaves of the whole plant were infected by 0–5% (excluding 0%). The heart leaf, tassel and corn ears were not infected.
2	Leaves of the whole plant were infected by 5–15% (excluding 5%), or the heart leaf, tassel and corn ears were infected by 0–5% (excluding 0%).
3	Leaves over the whole plant were infected by 15–25% (excluding 15%), or the heart leaf, tassel and corn ears were infected by 5–15% (excluding 5%).
4	Leaves over the whole plant were infected by 25–50% (excluding 25%), or the heart leaf, tassel and corn ears were infected by 15–25% (excluding 15%).
5	Leaves over the whole plant were infected over 50%, or the heart leaf, tassel and corn ears were infected over 25%.

## Data Availability

The data presented in this study are available on request from the corresponding author.
